# Author Correction: Generation of human islet cell type-specific identity genesets

**DOI:** 10.1038/s41467-024-46525-z

**Published:** 2024-03-22

**Authors:** Léon van Gurp, Leon Fodoulian, Daniel Oropeza, Kenichiro Furuyama, Eva Bru-Tari, Anh Nguyet Vu, John S. Kaddis, Iván Rodríguez, Fabrizio Thorel, Pedro L. Herrera

**Affiliations:** 1 Department of Genetic Medicine and Development, Faculty of Medicine, University of Geneva, rue Michel-Servet 1, 1211 Geneva, Switzerland; 2 Department of Genetics and Evolution, Faculty of Sciences, University of Geneva, Quai Ernest-Ansermet 30, 1211 Geneva, Switzerland; 3 Center for iPS Cell Research and Application (CiRA), Kyoto University, 53 Shogoin-Kawahara, Sakyo, 606-8507 Kyoto, Japan; 4 Department of Diabetes & Cancer Discovery Science, Arthur Riggs Diabetes and Metabolism Research Institute, Beckman Research Institute, City of Hope, 1500 E Duarte Rd, Duarte, CA 91010 USA

**Keywords:** Predictive markers, Transcriptomics, Diabetes

Correction to: *Nature Communications* 10.1038/s41467-022-29588-8, published online 19 April 2022

In the original version of this article the Figure Legends in the Supplementary Information were inadvertently omitted.

In addition, Supplementary Tables were missing, and are now included and cited in the main text as Supplementary data files 1–8.

The HTML has been updated to include a correct version of the Supplementary Information and Supplementary data files, and the PDF version of this Article has been corrected to include the correct citations of the Supplementary data files.

### Supplementary information

Description of Additional Supplementary Files

Updated Supplementary Information

Supplementary Data 1

Supplementary Data 2

Supplementary Data 3

Supplementary Data 4

Supplementary Data 5

Supplementary Data 6

Supplementary Data 7

Supplementary Data 8

