# Supplementary material for: Generation of human islet cell type-specific identity genesets
Source: Nat Commun. 2022 Apr 19;13:2020. doi: 10.1038/s41467-022-29588-8 (PMC9019032; doi:10.1038/s41467-022-29588-8)
Supplement: Supplementary file 1 — Supplementary Information [file 41467_2022_29588_MOESM1_ESM.pdf]

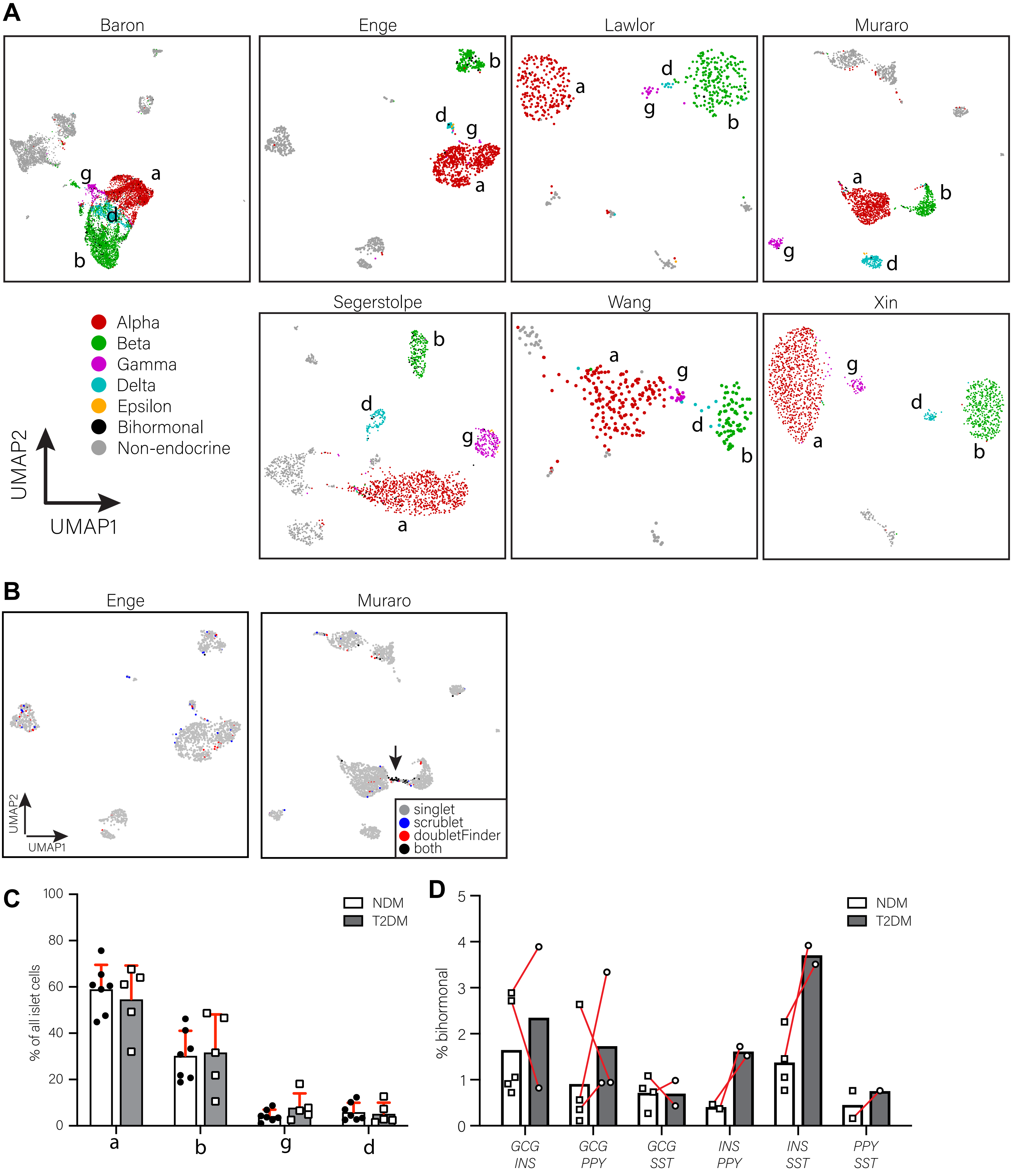


supplemental figure 1

**Supplemental figure 1. Generation and filtering of individual single-cell transcriptomics datasets.** A) UMAP dimension reduction plots of individual datasets after processing and filtering, indicated per dataset of each author. B) Examples of identification of doublets within datasets. Cells are color-coded based on which tool was used to identify them as doublets. The black arrow in the Muraro dataset indicates an area with a high concentration of cells that were indicated as doublets by both Scrublet and DoubletFinder. C) After final cell type allocation, endocrine cell type abundance was assessed for both NDM and T2DM cells, per dataset. There were no significant differences in abundance between NDM and T2DM cell types. D) Percentage of bi-hormonal cells, relative to its mono-hormonal counterparts. For example, the percentage of GCG-INS bi-hormonal cells is calculated over the total number of monohormonal cells that express either GCG or INS. Values were calculated per dataset in both non-diabetic (NDM) and type 2 diabetic (T2DM) populations, when applicable. Red lines connect NDM and T2DM data from the same dataset.

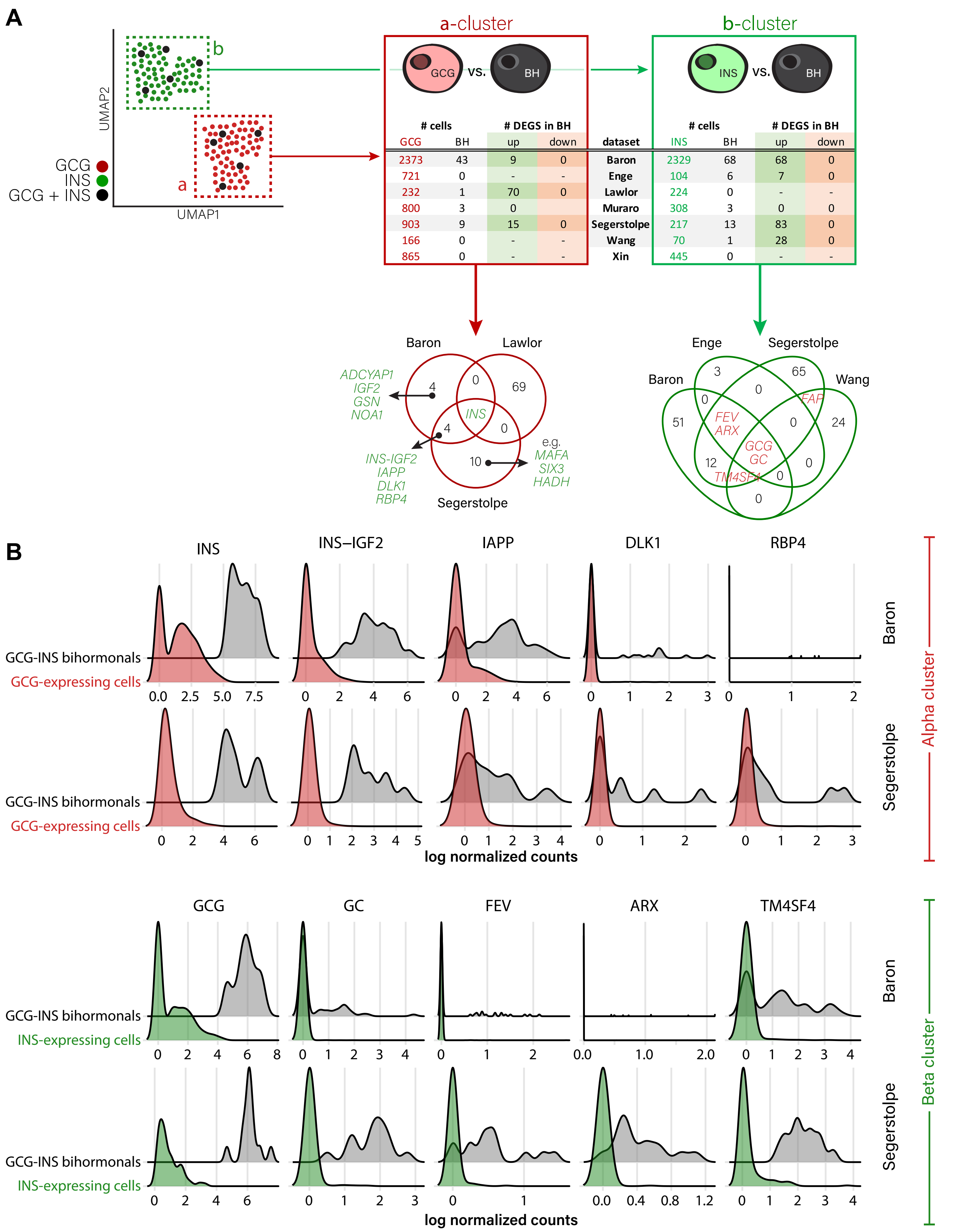


| a-cluster # cells GCG | a-cluster # cells BH | a-cluster # DEGS in BH up | a-cluster # DEGS in BH down | dataset | b-cluster # cells INS | b-cluster # cells BH | b-cluster # DEGS in BH up | b-cluster # DEGS in BH down |
|---|---|---|---|---|---|---|---|---|
| 2373 | 43 | 9 | 0 | Baron | 2329 | 68 | 68 | 0 |
| 721 | 0 | - | - | Enge | 104 | 6 | 7 | 0 |
| 232 | 1 | 70 | 0 | Lawlor | 224 | 0 | - | - |
| 800 | 3 | 0 | | Muraro | 308 | 3 | 0 | 0 |
| 903 | 9 | 15 | 0 | Segerstolpe | 217 | 13 | 83 | 0 |
| 166 | 0 | - | - | Wang | 70 | 1 | 28 | 0 |
| 865 | 0 | - | - | Xin | 445 | 0 | - | - |

supplemental figure 2

**Supplemental figure 2. Profiling of bihormonal cells.** A) Characterization of bi-hormonal cells within $\alpha$- and $\beta$-cell clusters. Within each cluster, differentially expressed genes were calculated between bi-hormonal cells and their mono-hormonal counterparts, as indicated in the table. Upregulated but no downregulated genes were found in the bi-hormonal cells. Per cell type cluster, upregulated genes were intersected to visualize genes that were upregulated in bihormonal cells in more than one dataset. B) Ridge plots depicting log normalized expression distribution in bihormonal cells vs. GCG expressing cells in the $\alpha$-cell cluster, and vs. INS expressing cells in the $\beta$-cell cluster, in the Baron and Segerstolpe datasets.

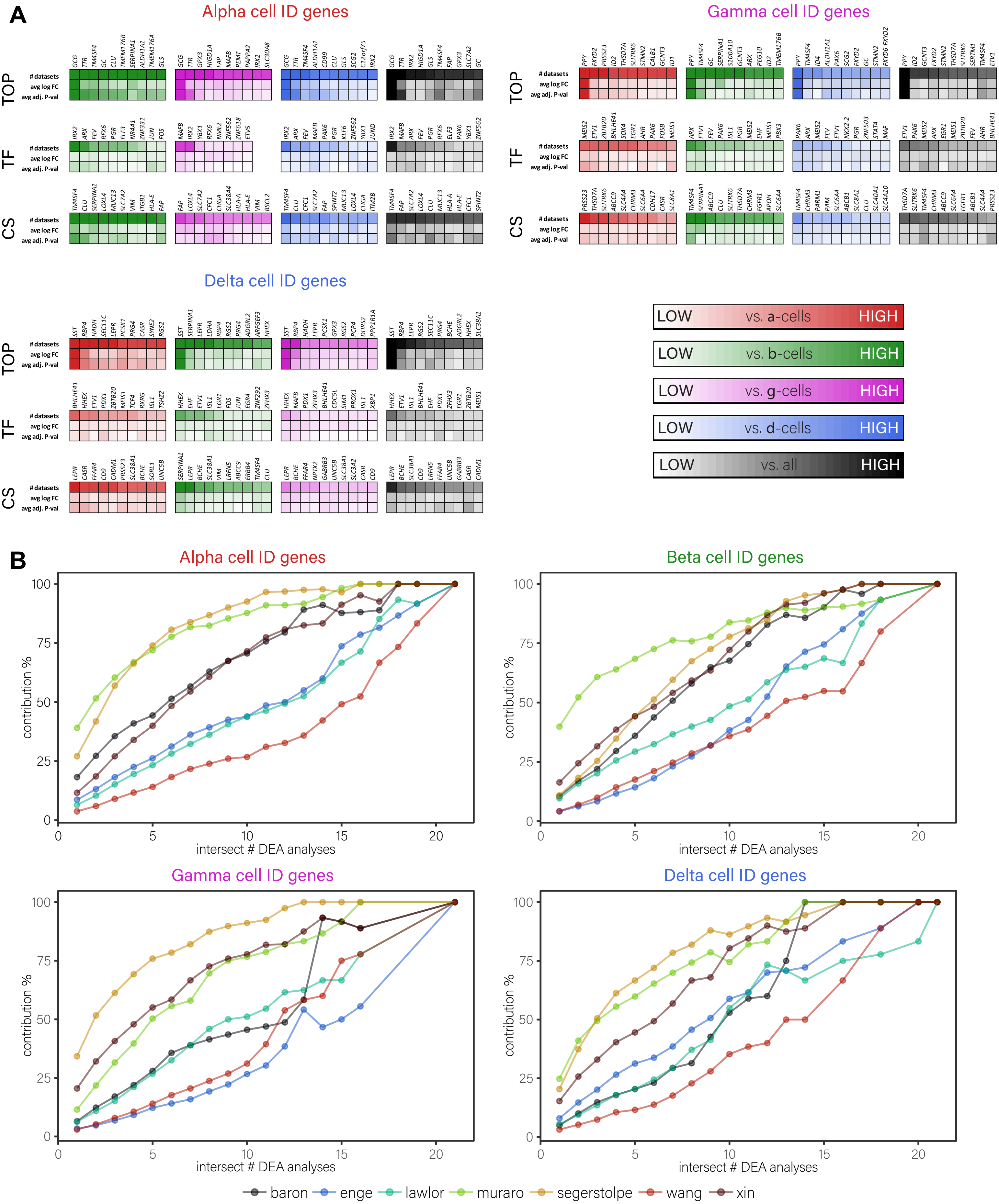


supplemental figure 3

**Supplemental figure 3. Defining identity genes for the different islet cell types.** A) Top differentially expressed genes (TOP), top transcription factors (TF) and top genes that encode cell-surface proteins (CS) that characterize $\alpha$-, $\gamma$- and $\delta$-cells, in direct comparisons (vs. $\alpha$-cells in red, vs. $\beta$-cells in green, vs. $\gamma$-cells in magenta and vs. $\delta$-cells in blue, respectively), and in a combined manner to define general identity genes (in black). Genes were order primarily on the # analyses in which they were found to be differentially expressed, then based on a rank score that comprised both the adjusted p-value and the log fold-change. Darker colors indicate a higher number of analyses, a higher log fold-change or a more significant adjusted p-value. B) Per cell type, the contribution of individual datasets to the total number of genes that are retrieved in at least the intersected number of analyses on the X-axis.

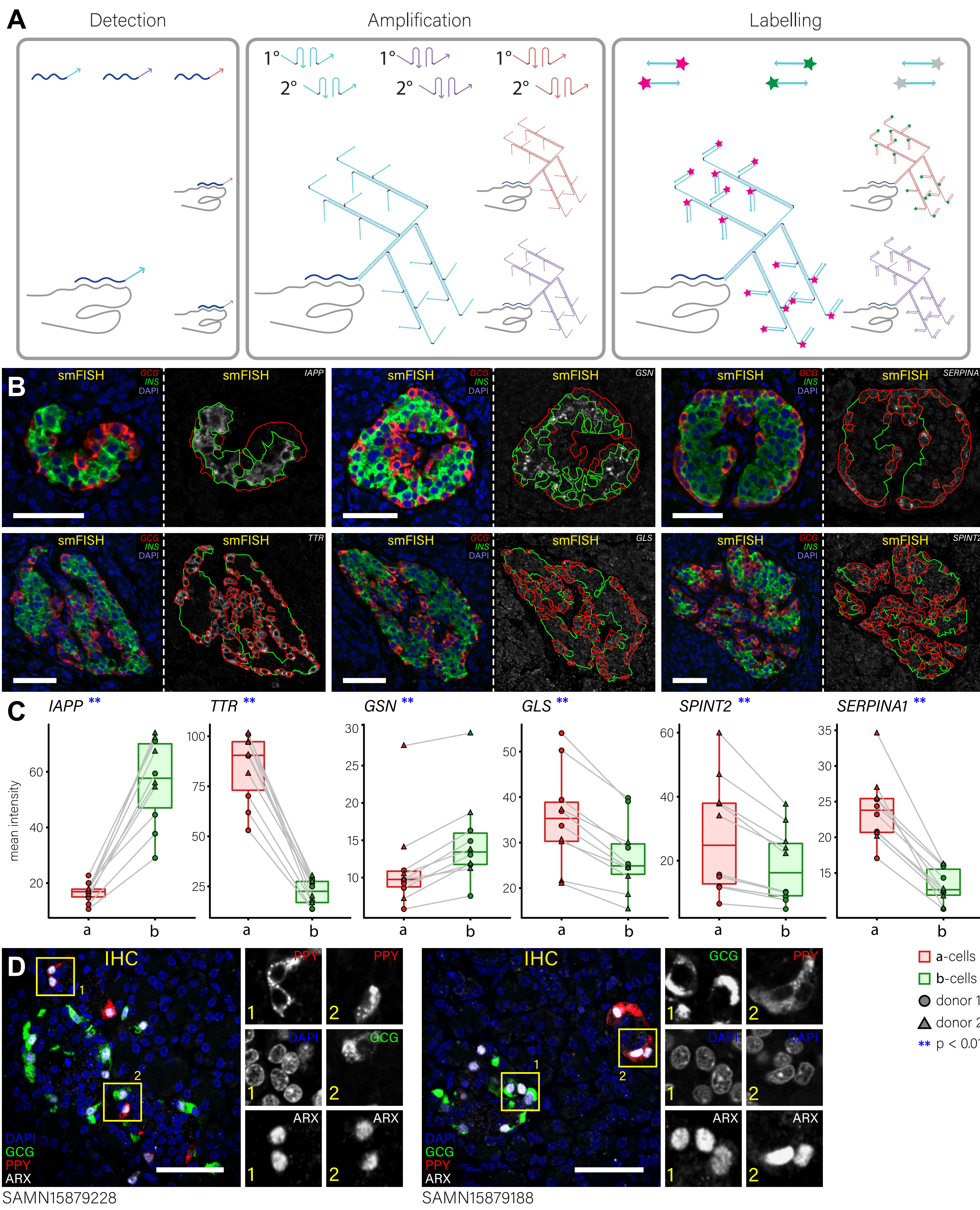


supplemental figure 4

**Supplemental figure 4. Orthologous validation of a- and b-cell identity markers.** A) methodology for MUSE-based multiplex smFISH. Between 9 and 22 probes were annealed per mRNA target. During the primary and secondary reactions, hairpin DNA structures unfold to build a amplification tree. During labelling, fluorescent dyes were coupled to these trees to amplify the signal of each bound mRNA probe. B) Representative images for each measured probe in human FFPE pancreatic tissue. Per probe, two images are provided: a composite smFISH image for *INS* and *GCG* with DAPI (left), and the monochromic image of the gene of interest, with outlines for $\alpha$- and $\beta$-cells derived from the GCG and INS signals, respectively (right). C) Measured mean intensities in paired $\alpha$- and $\beta$-cells from the same islet (2 donors, 5 islets per donor). Boxplot for $\alpha$-cells in red, for $\beta$-cells in green. Paired data connected via grey line. Donor 1 in circles, donor 2 in triangles. Statistical analysis performed as Wilcoxon singed rank test, ** = $p < 0.01$. D) Representative images of ARX staining in both $\alpha$- and $\gamma$-cells, in 2 independent donors (RRID: SAMN15879228 and SAMN15879188). Outlines illustrate specificity to $\alpha$- and $\gamma$-cells, and overlap with DAPI.

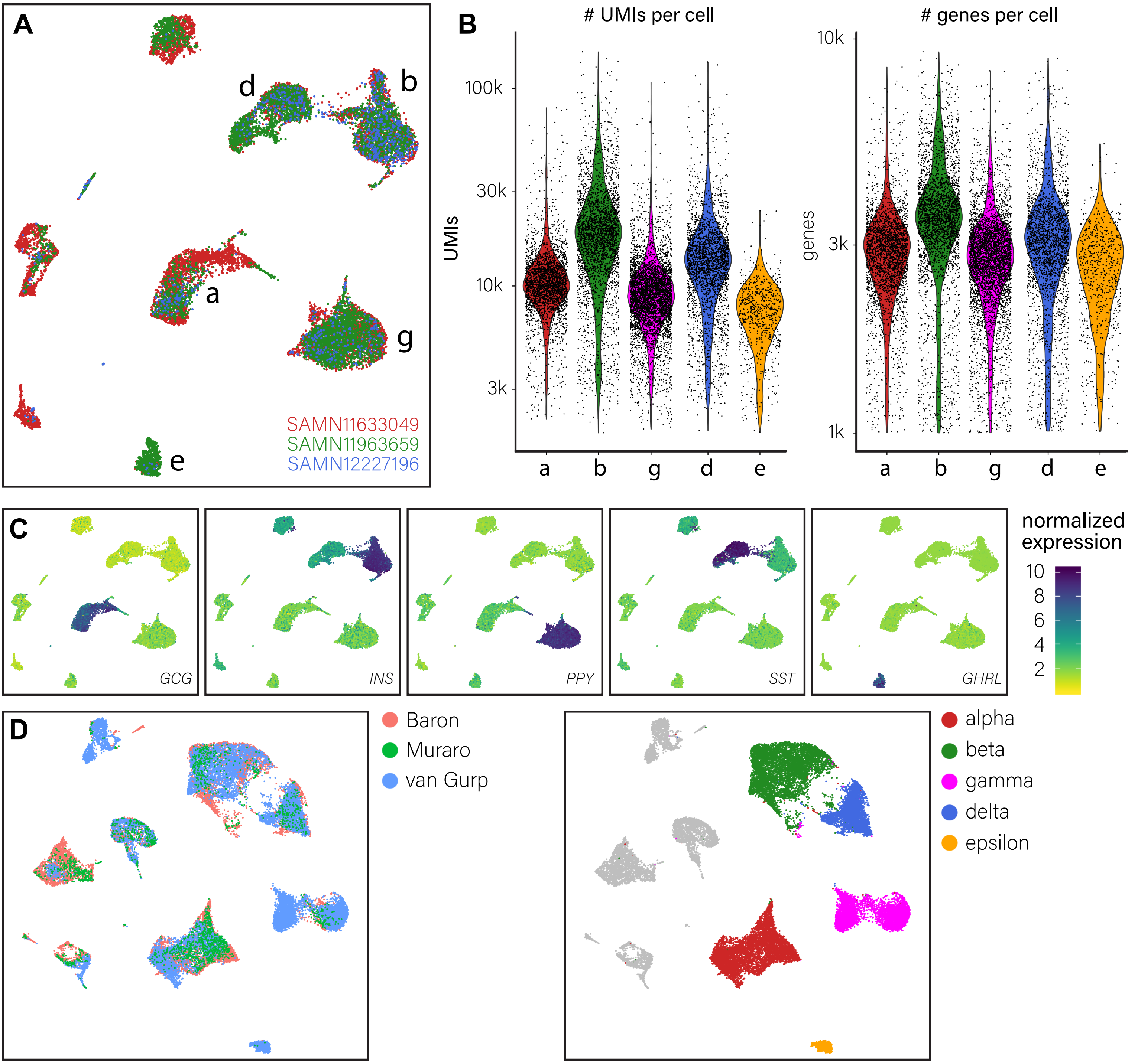


supplemental figure 5

**Supplemental figure 5. Quality assessment of our novel γ-, δ- and ε-cell enriched dataset.** A) Overlap of cells originating from different independent donors (RRID: SAMN11633049, SAMN11963659 and SAMN12227196). Cells are color-coded based on the donor they originate from. Cluster identities for endocrine cells are indicated by Greek letters. B) Data complexity of the novel dataset. For each cell type, the number of UMIs and genes per cell is indicated. C) UMAP dimensional reduction plots of the novel dataset, where cells are color-coded based on normalized hormone expression. D) Merged UMAPs of Baron, Muraro and our novel γ- δ- and ε-cell enriched datasets, color coded by either dataset origin or cell type.

**A**

| α-ID (IS# 13) 40 genes | β-ID (IS# 8) 127 genes | | | γ-ID (IS# 8) 45 genes | δ-ID (IS# 6) 75 genes | |
|---|---|---|---|---|---|---|
| ALDH1A1 | ABCC8 | KCNK16 | RPL4 | ABCB1 | ABCC9 | NLRP1 |
| B2M | AC099548.2 | LDHB | RPL5 | ABCC9 | ABI3BP | NPTX2 |
| CFC1 | ADCYAP1 | MAFA | RPL7 | AKAP12 | ADGRL2 | OPRD1 |
| CLU | ALCAM | MAFB | RPL7A | AMOTL1 | AKAP12 | PAPPA2 |
| CRYBA2 | ALDOA | MAP1B | RPS23 | AQP3 | AMIGO2 | PCP4 |
| CYSTM1 | ARG2 | MT-CO1 | RPS3 | ARX | ANK1 | PCSK1 |
| DPP4 | ARL6IP5 | MT-CO3 | RPS4X | C16orf45 | AQP3 | PDLIM4 |
| F10 | ATP2A3 | MT-CYB | RPS6 | CALB1 | ARFGEF3 | PKIB |
| FAP | C1orf127 | MT-ND1 | RRAGD | CARTPT | BAIAP3 | PLEKHB1 |
| FXYD3 | C1QL1 | MT-ND2 | SAMD11 | CHN2 | BCHE | PRG4 |
| FXYD5 | CADM1 | MT-ND3 | SCD | CHRM3 | BHLHE41 | PSIP1 |
| GC | CASR | MT-ND4 | SCD5 | CPB1 | C22orf42 | RASSF6 |
| GCG | CDKN1A | MTRNR2L1 | SCG3 | DPYSL3 | CADM1 | RBP4 |
| GLS | CDKN1C | MTRNR2L10 | SCGN | ETV1 | CALB1 | RGS2 |
| GPX3 | CNP | MTRNR2L12 | SELENOW | FGFR1 | CASR | S100A6 |
| HIGD1A | CYP2U1 | MTRNR2L6 | SERINC1 | FXYD2 | CBLN4 | SCD5 |
| HLA-A | CYYR1 | MTRNR2L8 | SLC30A8 | FXYD6-FXYD2 | CD9 | SEC11C |
| HLA-E | DBI | MTUS2 | SLC39A14 | GC | CPB1 | SERPINA1 |
| IRX2 | DHRS2 | MXRA7 | SLC6A6 | GCNT3 | CXADR | SLC17A6 |
| ITGB1 | DHRS7 | NECTIN3 | SORL1 | ID1 | DHRS2 | SLC38A1 |
| KCTD12 | DLK1 | NKX6-1 | STX1A | ID2 | DIRAS3 | SORL1 |
| LOXL4 | DNAJB9 | NPTX2 | SURF4 | ID4 | DPYSL3 | SST |
| MUC13 | EIF4A2 | P2RY1 | SUSD4 | INPP5F | EDN3 | SYNE2 |
| NAA20 | ELMO1 | PAPSS2 | SYT13 | MEIS2 | EHF | TENM3 |
| PALLD | ENO1 | PCSK1 | TGFBR3 | PAX6 | ERBB4 | TIMP2 |
| PAPPA2 | ENTPD3 | PDX1 | TIMP2 | PEG10 | ETV1 | TMSB4X |
| PCSK2 | ERO1B | PEBP1 | TMEM150C | PPY | EYS | TPPP3 |
| PEMT | FAM105A | PEMT | TMEM37 | PTP4A3 | F5 | UNC5B |
| PLCE1 | FAM159B | PERP | TNS1 | PXK | FAM102A | VIM |
| PLIN3 | FXYD2 | PFKFB2 | TPM3 | S100A10 | FFAR4 | VMP1 |
| RGS4 | G3BP1 | PFN2 | TSPAN1 | SCG2 | FRZB | |
| RNASEK | G6PC2 | PHACTR2 | TSPAN13 | SCGB2A1 | GABRB3 | |
| SLC7A2 | GAD2 | PLCXD3 | UCHL1 | SEMA3E | GPX3 | |
| SMIM24 | GLIS3 | PPP1R1A | VEGFA | SERTM1 | HADH | |
| SPINT2 | GNAS | PRDX1 | WARS | SLC4A4 | HHEX | |
| TM4SF4 | GSN | PRUNE2 | WSCD2 | SLC6A4 | ISL1 | |
| TMEM176A | HADH | PSAP | YWHAQ | SLITRK6 | LDHA | |
| TMEM176B | HERPUD1 | PTEN | | SPINK1 | LEPR | |
| TTR | HSP90AB1 | RBP4 | | SPOCK1 | LRFN5 | |
| VGF | HSPA8 | RGS16 | | STMN2 | MDK | |
| | IAPP | ROBO2 | | THSD7A | MLPH | |
| | IGF2 | RPL17 | | TM4SF4 | MS4A8 | |
| | INS | RPL23 | | TMEM176B | MTUS1 | |
| | INS-IGF2 | RPL24 | | TMEM47 | NCOA7 | |
| | ITPR3 | RPL3 | | TXNIP | NDRG4 | |

**B**

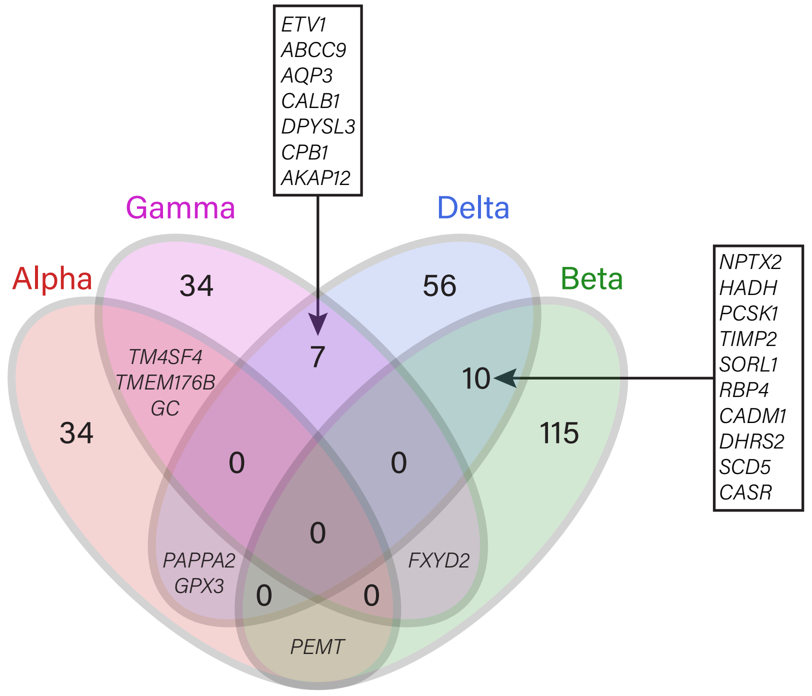

**Supplemental figure 6. Composition and overlap of the novel identity genesets.** A) Composition of the four identity genesets, $\alpha$-ID, $\beta$-ID, $\gamma$-ID and $\delta$-ID. B) Venn diagram showing genes overlapping between identity genesets.

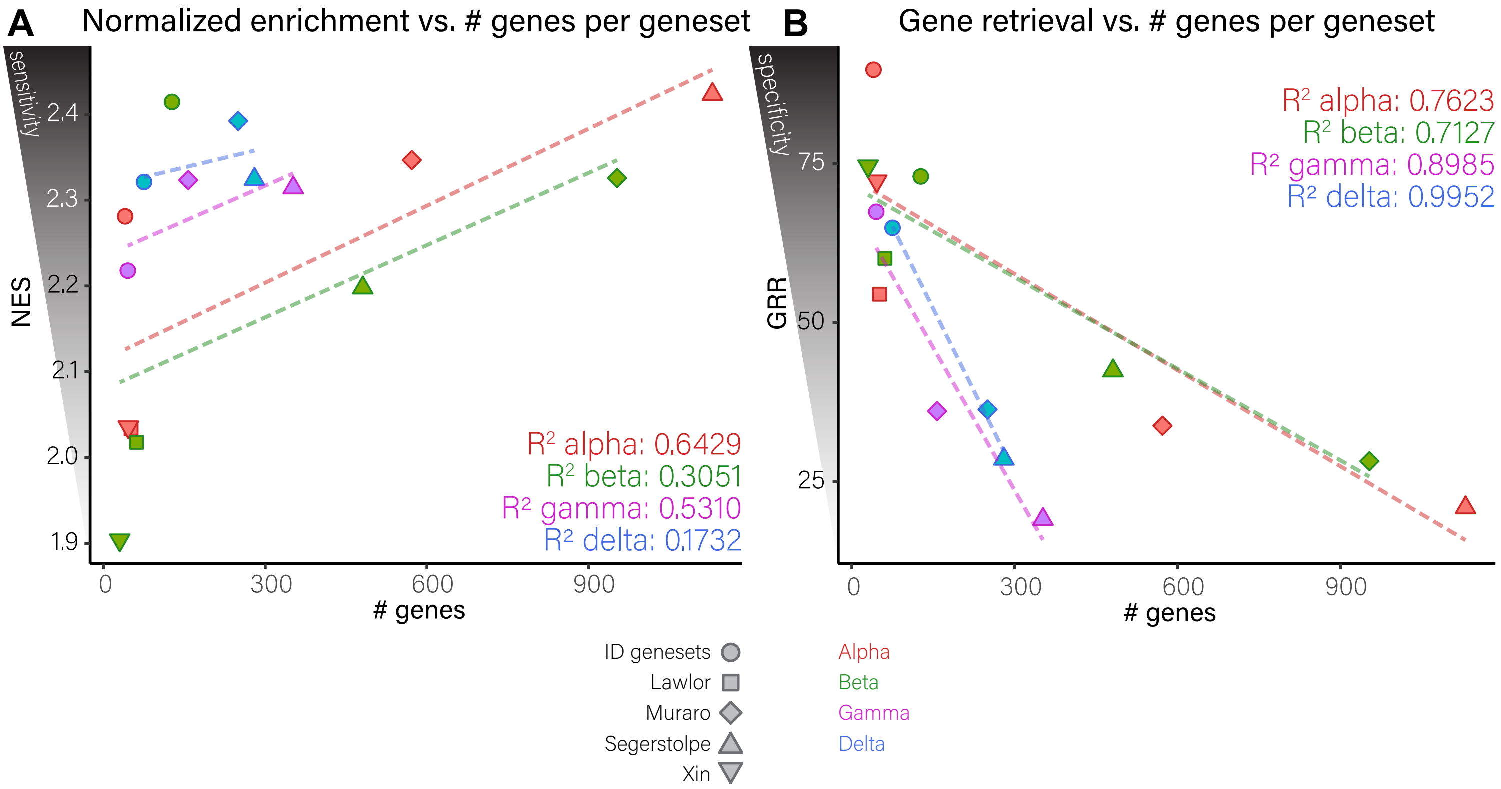
A
Normalized enrichment vs. # genes per geneset
sensitivity
NES
2.4
2.3
2.2
2.1
2.0
1.9
0
300
600
900
# genes
R² alpha: 0.6429
R² beta: 0.3051
R² gamma: 0.5310
R² delta: 0.1732
B
Gene retrieval vs. # genes per geneset
specificity
GRR
75
50
25
0
300
600
900
# genes
R² alpha: 0.7623
R² beta: 0.7127
R² gamma: 0.8985
R² delta: 0.9952
ID genesets
Lawlor
Muraro
Segerstolpe
Xin
Alpha
Beta
Gamma
Delta

**Supplemental figure 7. Relationships between the number of genes, normalized enrichment scores and gene retrieval rates in genesets.** A) The relationship between the number of genes in a dataset and the normalized enrichment score indicates a higher NES for genesets with more genes. Trendlines are color coded per cell type. B) The relationship between the number of genes in a dataset and the gene retrieval rate indicates a lower GRR for genesets with more genes. Trendlines are color coded per cell type. Shapes of individual points indicate the dataset of origin, while color indicates the associated cell type.

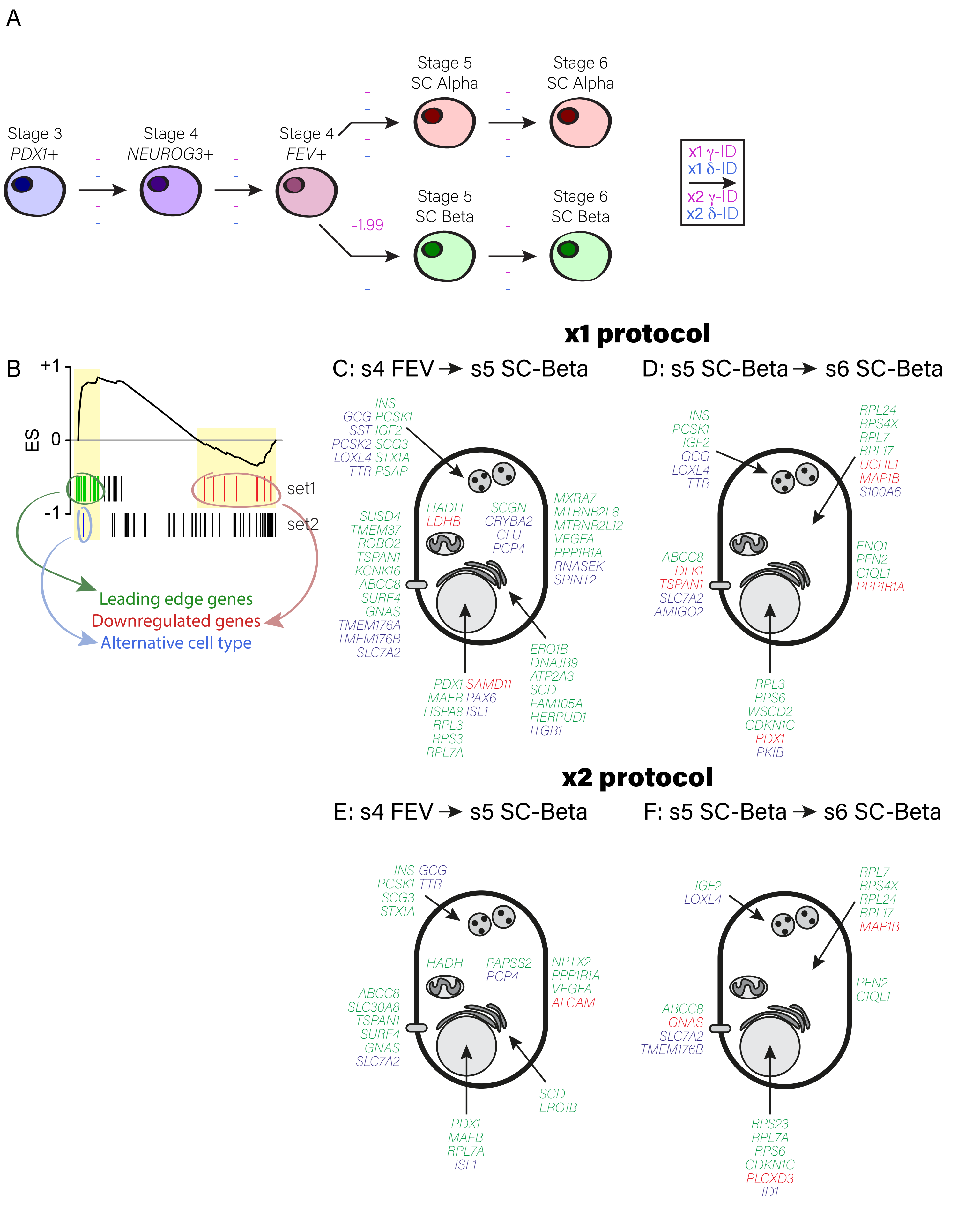


supplemental figure 8

**Supplemental figure 8. Deep profiling of SC-β cell identity changes**. A) Changes in gamma and delta cell identity in different human ES/iPS differentiation protocols. B) Definition of different categories of genes. Leading edge genes are the gene that are responsible for the initial push during GSEA, and are thus the genes responsible for the observed correlation between geneset and phenotype. Downregulated genes are genes that have negative rank scores that are thus responsible for a pull towards the alternative phenotype. Alternative cell type genes are genes that are observed in genesets for the other islet cell types, that are regulated within the leading-edge region of the GSEA. These genes regulate an acquisition of phenotypical changes related to another cell type than the one intended. C-F) Summary cartoons depicting leading edge, downregulated and alternative cell type genes for progression from stage 4 FEV expressing progenitors to stage 5 SC-β cells (C, E), and from stage 5 to stage 6 SC-β cells (D, F) in both the x1 (C, D) and x2 (E, F) differentiation protocols. Leading edge genes in green, downregulated genes in red and alternative cell type genes in blue.

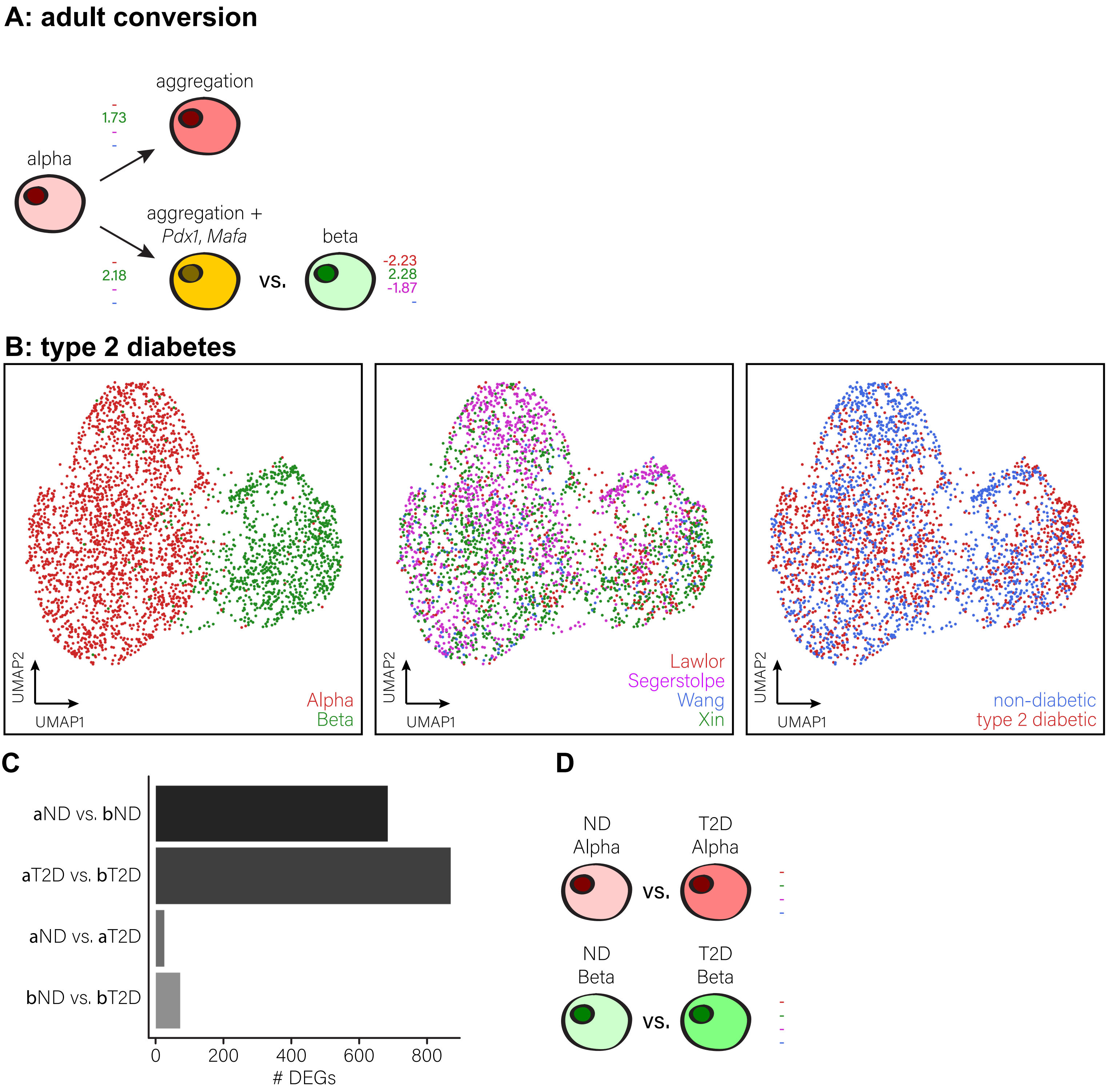
A: adult conversion
aggregation
alpha
1.73
aggregation +
Pdx1, Mafa
beta
2.18
vs.
-2.23
2.28
-1.87
B: type 2 diabetes
UMAP2
UMAP1
Alpha
Beta
Lawlor
Segerstolpe
Wang
Xin
non-diabetic
type 2 diabetic
C
aND vs. bND
aT2D vs. bT2D
aND vs. aT2D
bND vs. bT2D
0
200
400
600
800
# DEGs
D
ND
Alpha
T2D
Alpha
vs.
ND
Beta
T2D
Beta
vs.

**Supplemental figure 9. Additional assessment of identity regulation during adult cell type conversion and between non-diabetic and type 2 diabetic islet cells.** A) Changes in α-, β-, γ- and δ-cell identity were measured between states during adult cell type conversion. Values in red, green, magenta and blue indicate normalized enrichment scores from GSEA for α-ID, β-ID, γ-ID and δ-ID genesets, where positive values indicate a gain in identity and negative values indicate a loss of identity; ns = not significant (FDR 0.05 or higher). B) UMAP dimensional reduction of the combined dataset containing α- and β-cells fom the Lawlor, Segerstolpe, Wang and Xin datasets. Color codes indicate either cell type (left), dataset origin (middle) or diabetes status (right). C) The total number of differentially expressed genes between non-diabetic (ND) α- and β-cells, type 2 diabetic (T2D) α- and β- cells, ND and T2D α-cells and ND and T2D β-cells in the combined dataset. D) Changes in α-, β-, γ- and δ-cell identity were measured between ND and T2D α- and β-cells. No changes were found in any identity geneset.

| Dataset | | Donors | | Library preparation | | Median | Thresholds | | | Specific parameters | |
|---|---|---|---|---|---|---|---|---|---|---|---|
| First author | Last author | NDM | T2DM | Isolation | Processing | reads/cell | # counts | # genes | % mito | # var genes | # sig PCs |
| Baron | Yanai | 3 | 1 | inDrop | CEL-seq | 100k | UMIs 800 | 200 | 20 | 765 | 40 |
| Enge | Quake | 5 | - | FACS | SMARTseq2 | 950k | 100'000 | 2'500 | 15 | 832 | 16 |
| Lawlor | Stitzel | 5 | 3 | Fluidigm C1 | SMARTseq | 3'000k | 450'000 | 5'000 | 20 | 1'153 | 10 |
| Muraro | van Oudenaarden | 4 | - | FACS | CEL-seq2 | 90k | UMIs 2'000 | 350 | 25 | 1'069 | 40 |
| Segerstolpe | Sandberg | 6 | 4 | FACS | SMARTseq2 | 750k | 15'000 | 2'500 | 15 | 2'453 | 40 |
| Wang | Kaestner | 3 | 2 | Fluidigm C1 | SMARTseq2 | 2'200k | 400'000 | 2'500 | 15 | 1'411 | 11 |
| Xin | Gromada | 12 | 6 | Fluidigm C1 | SMARTseq2 | 950k | 350'000 | 4'500 | 15 | 1'371 | 12 |

supplemental table 1

**Supplemental table 1:** Details regarding the original datasets

| Dataset | Health | Sex | Age | BMI |
|---|---|---|---|---|
| Baron | NDM | M | 20-30 | 21.5 |
| Baron | NDM | F | 50-60 | 21.1 |
| Baron | NDM | M | 30-40 | 27.5 |
| Baron | T2DM | F | 50-60 | 29.9 |
| Enge | NDM | M | 20-30 | 28.4 |
| Enge | NDM | M | 20-30 | 24.8 |
| Enge | NDM | F | 30-40 | 29.5 |
| Enge | NDM | F | 40-50 | 23.8 |
| Enge | NDM | M | 50-60 | 27.3 |
| Fang | NDM | M | 20-30 | 20.6 |
| Fang | NDM | M | 20-30 | 22.8 |
| Fang | NDM | F | 30-40 | 34.4 |
| Fang | NDM | M | 50-60 | 22.0 |
| Fang | NDM | M | 20-30 | 30.8 |
| Fang | NDM | M | 40-50 | 34.6 |
| Fang | T2DM | M | 50-60 | 39.3 |
| Fang | T2DM | M | 60-70 | 28.1 |
| Fang | T2DM | M | 50-60 | 35.6 |
| Lawlor | NDM | M | 20-30 | 33.0 |
| Lawlor | T2DM | M | 50-60 | 35.8 |
| Lawlor | NDM | F | 50-60 | 22.0 |
| Lawlor | NDM | M | 20-30 | 23.0 |
| Lawlor | NDM | M | 30-40 | 55.0 |
| Lawlor | NDM | F | 50-60 | 26.6 |
| Lawlor | T2DM | F | 50-60 | 29.8 |
| Lawlor | T2DM | F | 40-50 | 43.0 |
| Muraro | NDM | M | 50-60 | 26.0 |
| Muraro | NDM | M | 20-30 | 22.0 |
| Muraro | NDM | F | 40-50 | 26.0 |
| Muraro | NDM | M | 50-60 | 25.0 |
| Segerstolpe | NDM | M | 40-50 | 30.8 |
| Segerstolpe | NDM | M | 20-30 | 24.7 |
| Segerstolpe | NDM | M | 20-30 | 21.5 |
| Segerstolpe | NDM | F | 40-50 | 35.0 |
| Segerstolpe | NDM | M | 20-30 | 32.9 |
| Segerstolpe | NDM | M | 20-30 | 31.8 |
| Segerstolpe | T2DM | M | 50-60 | 24.0 |
| Segerstolpe | T2DM | F | 30-40 | 39.6 |
| Segerstolpe | T2DM | M | 50-60 | 34.4 |
| Segerstolpe | T2DM | F | 50-60 | 29.8 |
| van Gurp | NDM | M | 40-50 | 38.8 |
| van Gurp | NDM | M | 50-60 | 32.6 |
| van Gurp | NDM | M | 50-60 | 31.8 |
| van Gurp | NDM | F | 10-20 | 32.4 |
| van Gurp | NDM | M | 20-30 | 24.8 |
| Wang | NDM | M | 50-60 | 29.1 |
| Wang | T2DM | M | 50-60 | 24.0 |
| Wang | T2DM | F | 30-40 | 39.3 |
| Wang | NDM | M | 20-30 | 39.0 |
| Wang | NDM | F | 30-40 | 45.2 |
| Xin | NDM | M | 20-30 | 21.0 |
| Xin | NDM | F | 30-40 | 19.0 |
| Xin | NDM | F | 20-30 | 24.5 |
| Xin | NDM | F | 50-60 | 24.1 |
| Xin | NDM | M | 20-30 | 31.8 |
| Xin | NDM | M | 60-70 | 26.0 |
| Xin | NDM | M | 20-30 | 23.4 |
| Xin | NDM | M | 60-70 | 27.3 |
| Xin | NDM | F | 20-30 | 25.4 |
| Xin | NDM | M | 40-50 | 31.7 |
| Xin | NDM | F | 30-40 | 28.0 |
| Xin | NDM | M | 50-60 | 22.8 |
| Xin | T2DM | M | 50-60 | 24.0 |
| Xin | T2DM | F | 30-40 | 39.6 |
| Xin | T2DM | F | 50-60 | 29.9 |
| Xin | T2DM | F | 40-50 | 43.1 |
| Xin | T2DM | M | 40-50 | 43.7 |
| Xin | T2DM | M | 50-60 | 24.4 |
| Xin2 | NDM | F | 30-40 | 24.9 |
| Xin2 | NDM | M | 20-30 | 24.8 |
| Xin2 | NDM | M | 50-60 | 30.0 |
| Xin2 | NDM | M | 50-60 | 22.0 |
| Xin2 | NDM | M | 40-50 | 26.5 |
| Xin2 | NDM | F | 30-40 | 22.9 |
| Xin2 | NDM | M | 30-40 | 23.6 |
| Xin2 | NDM | M | 20-30 | 30.8 |
| Xin2 | NDM | M | 40-50 | 26.2 |
| Xin2 | NDM | F | 50-60 | 23.5 |
| Xin2 | NDM | F | 40-50 | 23.0 |
| Xin2 | NDM | M | 50-60 | 21.2 |

supplemental table 2

**Supplemental table 2:** Donor specific information for all datasets used in this study

| | # cells | Doublet removal | | | # Singlets | | non-diabetic # | | | | | type 2 diabetic # | | | | |
|---|---|---|---|---|---|---|---|---|---|---|---|---|---|---|---|---|
| | initial | DF | Scr | both | total | islet | α | β | γ | δ | ε | α | β | γ | δ | ε |
| Baron | 9'005 | 329 | 123 | 11 | 8'542 | 5'766 | 1'974 | 1'814 | 163 | 449 | 10 | 434 | 659 | 89 | 172 | 2 |
| Enge | 1'408 | 42 | 34 | 4 | 1'328 | 970 | 730 | 200 | 10 | 25 | 5 | - | - | - | - | - |
| Lawlor | 560 | 14 | 4 | 5 | 537 | 484 | 138 | 134 | 10 | 8 | 1 | 95 | 90 | 5 | 3 | 0 |
| Muraro | 2'116 | 38 | 17 | 40 | 2'021 | 1'439 | 847 | 313 | 99 | 177 | 3 | - | - | - | - | - |
| Segerstolpe | 2'166 | 63 | 33 | 14 | 2'056 | 1'459 | 510 | 147 | 68 | 55 | 5 | 431 | 71 | 122 | 49 | 1 |
| Wang | 284 | 4 | 7 | 5 | 268 | 262 | 73 | 40 | 3 | 4 | 0 | 96 | 31 | 11 | 4 | 0 |
| Xin | 1'503 | 40 | 1 | 6 | 1'456 | 1'422 | 341 | 174 | 24 | 25 | 0 | 524 | 271 | 42 | 21 | 0 |

| | non-diabetic % | | | | | type 2 diabetic % | | | | |
|---|---|---|---|---|---|---|---|---|---|---|
| | α | β | γ | δ | ε | α | β | γ | δ | ε |
| Baron | 44.8 | 41.1 | 3.7 | 10.2 | 0.2 | 32.0 | 48.6 | 6.6 | 12.7 | 0.1 |
| Enge | 75.3 | 20.6 | 1.0 | 2.6 | 0.5 | | | | | |
| Lawlor | 47.4 | 46.0 | 3.4 | 2.7 | 0.3 | 49.2 | 46.6 | 2.6 | 1.6 | 0.0 |
| Muraro | 58.9 | 21.8 | 6.9 | 12.3 | 0.2 | | | | | |
| Segerstolpe | 65.0 | 18.7 | 8.7 | 7.0 | 0.6 | 63.9 | 10.5 | 18.1 | 7.3 | 0.1 |
| Wang | 60.8 | 33.3 | 2.5 | 3.3 | 0.0 | 67.6 | 21.8 | 7.7 | 2.8 | 0.0 |
| Xin | 60.5 | 30.9 | 4.3 | 4.4 | 0.0 | 61.1 | 31.6 | 4.9 | 2.4 | 0.0 |
| | | | | | | | | | | |
| Average | 58.9 | 30.4 | 4.4 | 6.1 | 0.3 | 54.8 | 31.8 | 8.0 | 5.4 | 0.1 |
| SD | 10.3 | 10.6 | 2.6 | 3.9 | 0.2 | 14.5 | 16.2 | 6.0 | 4.7 | 0.1 |

supplemental table 3

**Supplemental table 3:** The numbers and percentages of cells in each public dataset used in thsi study

supplemental table 4

| cell-type | gene | cell-type specific function | specificity to cell-type | pmid | pmid | pmid |
|---|---|---|---|---|---|---|
| alpha cells | GCG | secreted to raise blood glucose levels, paracrine signaling | glucagon is uniquely expressed in alpha cells in the pancreatic islets | 25850661 | 27044683 | 15437813 |
| alpha cells | TTR | regulates glucagon expression, more TTR expressing cells in T2D | alpha cell specific under non-diabetic conditions | **this manuscript** | 23108050 | 18825272 |
| alpha cells | IRX2 | involved in alpha cell specific gene expression, critical for iPS alpha cell development | co-localizes with glucagon expression in adult islets | 17032746 | 32382023 | |
| alpha cells | HIGD1A | may protect cells from oxidative stress | Expressed in alpha cells, but not beta cells | 16815968 | 31089410 | |
| alpha cells | GLS | produces glutamate, which enhances glucagon secretion | mainly present in alpha cells | **this manuscript** | 15089745 | 26740469 |
| alpha cells | TM4SF4 | inhibits alpha and beta cell fate acquisition during development | allows FACS based enrichment for adult human alpha cells | 27693023 | 21750032 | |
| alpha cells | FAP | serine protease involved in paracrine signaling | co-expresses with DPP4 and GCG | 25361590 | | |
| alpha cells | SLC7A2 | involved in alpha cell proliferation and glucagon secretion | more highly expressed than in beta cells | https://doi.org/10.2337/db19-198-OR | | |
| alpha cells | GC | - | alpha cell specific in gene transcription and chromatin accessibility | 31120862 | 33719063 | |
| alpha cells | PCSK2 | involved in glucagon processing | in humans PCSK2 is specific for alpha cells | 32291281 | | |
| alpha cells | CLU | immunoprotective, may impact paracrine beta cell proliferation | coexpression with glucagon, alpha cell ultrastructure | 10396021 | 11793021 | |
| alpha cells | SPINT2 | - | smFISH stronger expression in alpha- than beta-cells | **this manuscript** | | |
| alpha cells | SERPINA1 | - | smFISH stronger expression in alpha- than beta-cells | **this manuscript** | | |
| alpha cells | ARX | ARX required for alpha cell development, required for ID maintenance | strong expression colocalization in alpha- and gamma-cells IHC | **this manuscript** | 14561778 | 23785486 |
| beta cells | INS | secreted to lower blood glucose levels, paracrine signaling | insulin is uniquely expressed in beta cells in the pancreatic islets | 19871205 | 14365544 | 7019246 |
| beta cells | IAPP | cosecreted with insulin, slows gastric emptying and promotes satiety | IHC costaining with INS, GCG, PPY and SST shows b-cell specificity | **this manuscript** | 3276206 | 10341286 |
| beta cells | G6PC2 | beta cell specific KO shows involvement in fasting blood glucose | Coexpresses with insulin, not glucagon IHC | 32213654 | 12861077 | |
| beta cells | ADCYAP1 | providex protection against beta cell death, may be involved in proliferation | strong coexpression with both insulin and glucagon IHC | 12716746 | 12686458 | |
| beta cells | ERO1B | involved in insulin protein folding | colocalization between insulin and ERO1B in IHC | 20308425 | 21540283 | |
| beta cells | DLK1 | together with MEG3 involved in cytokine-mediated sensitivity to oxidative stress | FACS shows beta cells can be distiguished as DLK1 high and low | 30084829 | 24049066 | |
| beta cells | NPTX2 | - | colocalization with INS, not with GCG in IHC | https://doi.org/10.32473/ufjur.v21i2.108732 | | |
| beta cells | GSN | protects beta cells from apoptosis, involved in insulin secretion | smFISH stronger expression in alpha- than beta-cells | **this manuscript** | 17192468 | |
| beta cells | INS-IGF2 | - | specifically co-expressed with insulin in b-cells | 26220792 | | |
| beta cells | HADH | hyperinsulinism in beta cell specific Hadh-KO mice | specifically colocalizes with INS in beta cell grafts IHC | 26953163 | https://doi.org/10.2337/db18-1824-P | |
| beta cells | IGF2 | involved in beta cell mass and function maintenance during stress | - | 26384384 | | |
| beta cells | PEBP1 | inhibits beta cell proliferation | RKIP colocalizes with beta and gamma cells in IHC | 15349122 | | |
| beta cells | PFKFB2 | PFKFB2 expression correlates with insulin secretion | - | 25662186 | | |
| beta cells | SLC30A8 | required for in vivo insulin release | ZNT8 colocalizes with INS not GCG in human sections | 19542200 | | |
| beta cells | ABCC8 | encodes SUR1 critical component of Ca2+ channel, deletion leads to ID loss | - | 16416420 | 28550109 | |
| beta cells | SCGN | involved in beta cell specification during development, | protein expressed in alpha and beta cells IHC | 29910119 | 29702679 | |
| gamma cells | PPY | decreases appetite, promotes satiety | localization of PPY in pancreatic islets, distinct from A-, B- and D-cells | 782992 | 15068960 | |
| gamma cells | PAX6 | PP cells do not develop in PAX4/PAX6 double KO, but appear in single PAX4 KO | expressed in all islet cell-types. Colocalization with PP in IHC | 9163426 | 10704887 | |
| gamma cells | ARX | ARX/PAX4 axis defines gamma cells together with alpha-cells | strong expression colocalization in alpha- and gamma-cells | **this manuscript** | 26319183 | |
| delta cells | SST | paracrine inhibition of alpha- and beta-cell sectetion of GC and INS | secreted hormone that in the pancreas is uniquely produced by delta cells | 1355587 | | |
| delta cells | LEPR | - | smFISH on human oct embedded pancreas shows colocalization with SST | 27864352 | | |
| delta cells | HHEX | critical player in driving and maintaining delta cell identity | HHEX colocalizes with SST, not with INS and GCG in IHC | 24736842 | | |
| delta cells | CD9 | - | allows enrichment for delta cells during FACS icw 2B4 and 2D12 antibodies | **this manuscript** | | |
| delta cells | PCSK1 | - | PCSK1 is upregulated and colocalizes with SST in ARX-KO hESC derived endocrine cells | 26633894 | | |
| delta cells | FFAR4 | regulates somatostatin secretion | strong colocalization of Ffar4 with Sst in mice IHC | 24663807 | | |

**Supplemental table 4:** literature based validation of identity genes

| | total cells | α | % | β | % | γ | % | δ | % | ε | % | BH | % | other | % |
|---|---|---|---|---|---|---|---|---|---|---|---|---|---|---|---|
| Donor 1 | 7'788 | 1'880 | 24.1 | 1'225 | 15.7 | 1'786 | 22.9 | 698 | 9.0 | 48 | 0.6 | 139 | 1.8 | 2'012 | 25.8 |
| Donor 2 | 6'764 | 521 | 7.7 | 1'167 | 17.3 | 2'096 | 31.0 | 1'454 | 21.5 | 610 | 9.0 | 228 | 3.4 | 688 | 10.2 |
| Donor3 | 935 | 91 | 9.7 | 375 | 40.1 | 200 | 21.4 | 163 | 17.4 | 8 | 0.9 | 38 | 4.1 | 60 | 6.4 |
| | 15'487 | 2'492 | 16.1 | 2'767 | 17.9 | 4'082 | 26.4 | 2'315 | 14.9 | 666 | 4.3 | 405 | 2.6 | 2'760 | 17.8 |

α = alpha cells
β = beta cells
γ = gamma cells
δ = delta cells
ε = epsilon cells
BH = bihormonal cells

**Supplemental table 5:** Cell numbers and percentages for the gamma/delta/epsilon cell enriched dataset presented in this study

| | duct -> neurog3 | neurog3 -> fev | fev -> alpha | fev -> beta |
|---|---|---|---|---|
| α-ID | | | 2.22 | |
| β-ID | | | | 1.85 |
| γ-ID | | | | |
| δ-ID | | | | |
| Lawlor α | | | 2.20 | |
| Lawlor β | -1.66 | | | 1.75 |
| Lawlor γ | | | | |
| Lawlor δ | | | | |
| Muraro α | 1.57 | 2.55 | 1.52 | |
| Muraro β | -1.53 | 1.81 | | |
| Muraro γ | 1.82 | 2.02 | | -1.70 |
| Muraro δ | 1.42 | 2.18 | | |
| Segerstolpe α | 1.43 | 1.89 | 1.54 | |
| Segerstolpe β | | 1.76 | | 1.54 |
| Segerstolpe γ | 1.77 | 2.11 | | |
| Segerstolpe δ | 1.53 | 1.92 | | |
| Xin α | | | 1.98 | |
| Xin β | | | | |
| Xin γ | | | | |
| Xin δ | | | | |

**Supplemental table 6:** enrichment scores for pancreas development comparing our idenrtity genesets to genesets derived from the original studies
