# Supplementary material for: Generation of human islet cell type-specific identity genesets
Source: Nat Commun. 2022 Apr 19;13:2020. doi: 10.1038/s41467-022-29588-8 (PMC9019032; doi:10.1038/s41467-022-29588-8)
Supplement: Supplementary file 3 — Reporting summary [file 41467_2022_29588_MOESM3_ESM.pdf]

nature portfolio

Corresponding author(s): Pedro L. Herrera

Last updated by author(s): March 2, 2022

Reporting Summary

Nature Portfolio wishes to improve the reproducibility of the work that we publish. This form provides structure for consistency and transparency in reporting. For further information on Nature Portfolio policies, see our Editorial Policies and the Editorial Policy Checklist.

Statistics

For all statistical analyses, confirm that the following items are present in the figure legend, table legend, main text, or Methods section.

n/a	Confirmed	
☐	☒	The exact sample size (*n*) for each experimental group/condition, given as a discrete number and unit of measurement
☐	☒	A statement on whether measurements were taken from distinct samples or whether the same sample was measured repeatedly
☐	☒	The statistical test(s) used AND whether they are one- or two-sided<br>*Only common tests should be described solely by name; describe more complex techniques in the Methods section.*
☐	☒	A description of all covariates tested
☒	☐	A description of any assumptions or corrections, such as tests of normality and adjustment for multiple comparisons
☐	☒	A full description of the statistical parameters including central tendency (e.g. means) or other basic estimates (e.g. regression coefficient) AND variation (e.g. standard deviation) or associated estimates of uncertainty (e.g. confidence intervals)
☐	☒	For null hypothesis testing, the test statistic (e.g. *F*, *t*, *r*) with confidence intervals, effect sizes, degrees of freedom and *P* value noted<br>*Give P values as exact values whenever suitable.*
☒	☐	For Bayesian analysis, information on the choice of priors and Markov chain Monte Carlo settings
☒	☐	For hierarchical and complex designs, identification of the appropriate level for tests and full reporting of outcomes
☒	☐	Estimates of effect sizes (e.g. Cohen's *d*, Pearson's *r*), indicating how they were calculated

Our web collection on statistics for biologists contains articles on many of the points above.

Software and code

Policy information about availability of computer code

Data collection

Single-read RNA sequencing data (Lawlor et al., Segerstolpe et al., Wang et al. and Xin et al. were downloaded as SRA files from the NCBI sequence read archive, and then transformed to fastq files using fastq-dump version 2.8.2. Paired-end RNA sequencing data (Enge et al., Baron et al. and Muraro et al. were downloaded as fastq files from the European Nucleotide Archive (ENA).

The following datasets were downloaded as processed data: matrices from 6x non-diabetic and 3x type 2 diabetic donors from Fang et al., matrices from 12x non-diabetic donors from Xin2 et al., the Seurat subset Rdata object from van Gurp et al., matrices from stage 3-6 for protocols X1 and X2 from Veres et al and all samples for sorted alpha, sorted beta, alpha-GFP and alpha-PM conditions from Furuyama et al.

For the gamma-delta-epsilon enriched dataset, gamma/epsilon- and delta-fraction of each donor were processed as independent experiments using the Chromium single cell gene expression protocol v3 (10x Genomics).

Data analysis

Seurat objects were generated for individual single cell datasets using Seurat version 2.3.4 (Baron, Enge, Fang, Lawlor, Muraro, Segerstolpe, Wang, Xin and Xin2 datasets). Seurat objects for the gamma-delta-epsilon enriched dataset, and the Veres dataset, were created using Seurat version 3.1.0. Data integration of the Lawlor, Segerstolpe, Wang and Xin datasets was performed using the RunMultiCCA tool included in Seurat 2.3.4. Data integration per donor of the gamma-delta-epsilon enriched dataset, and from the combined datasets of Baron, Muraro and the gamma-delta-epsilon enriched dataset, was performed using the IntegrateData functionality included in Seurat 3.1.0.

For the gamma-delta-epsilon enriched dataset, and the Baron, Enge, Fang, Lawlor, Muraro, Segerstolpe, Veres, Wang, Xin and Xin2 datasets, doublets were detected in each dataset using two independent tools: DoubletFinder v2.0.2 and Scrublet v0.2.1.

For Seurat objects created under seurat 2.3.4, clustering was assessed using clustree version 0.4.0.

Cell type allocation was based on both graph-based clustering and hormone expression, where cells assigned to a given population were never allowed to express more than a single hormone, or a hormone that is not commonly associated with that cell-type.

Differential expression in UMI datasets was calculated using the included "negbinom" test. In non-UMI datasets, differential expression was calculated using the MAST test instead. Gene annotation was based on collected information from the Compartments database and the human protein atlas.

Robust identity markers were identified by integrating differential expression results of pairwise analyses in 7 datasets, so that for each gene, the number of analyses in which it was identified was known, together with an average log fold change and Bonferroni corrected p-value. Threshold determination of which genes to include in the final genesets was based on geneset enrichment analysis (GSEA), which was performed using the GSEA java app version 4.0.1, available from the molecular signatures database website (http://www.gsea-msigdb.org/gsea/downloads.jsp).

Unless otherwise noted, comparisons between distinct samples were tested for significance using a two-sided Wilcoxon ranked sum test.

All code used to generate data in this manuscript are available upon justified request. Code used to share data is available on GitLab (https://gitlab.com/hirn-apps/scpancmeta).

For manuscripts utilizing custom algorithms or software that are central to the research but not yet described in published literature, software must be made available to editors and reviewers. We strongly encourage code deposition in a community repository (e.g. GitHub). See the Nature Portfolio guidelines for submitting code & software for further information.

Data

Policy information about availability of data

All manuscripts must include a data availability statement. This statement should provide the following information, where applicable:

- Accession codes, unique identifiers, or web links for publicly available datasets
- A description of any restrictions on data availability
- For clinical datasets or third party data, please ensure that the statement adheres to our policy

Genesets can be downloaded from MSigDB (https://www.gsea-msigdb.org/gsea/msigdb/index.jsp). All data in this manuscript can be accessed through an R-shiny based web app (https://rapps.hirnetwork.org/scPancMeta). Our gamma-delta-epsilon enriched datasets can be accessed through the Gene Expression Omnibus (accession nr. GSE150724). The following datasets were downloaded as SRA files from the NCBI sequence read archive: Lawlor (SRP075970 [https://www.ncbi.nlm.nih.gov/geo/query/acc.cgi?acc=GSE86473]), Segerstolpe (ERP017126 [https://www.omicsdi.org/dataset/arrayexpress-repository/E-MTAB-5061]), Wang (SRP076307) and Xin (SRP075377). The Baron (PRJNA328774), Enge (PRJNA322355) and Muraro (PRJNA337935) datasets were downloaded as fastq files from the European Nucleotide Archive (ENA). The following datasets were downloaded as processed data: Fang (GSE101207 [https://www.ncbi.nlm.nih.gov/geo/query/acc.cgi?acc=GSE101207]), Xin (GSE114297 [https://www.ncbi.nlm.nih.gov/geo/query/acc.cgi?acc=GSE114297]), van Gurp (GSE132364 [https://www.ncbi.nlm.nih.gov/geo/query/acc.cgi?acc=GSE132364]), Veres (GSE114412 [https://www.ncbi.nlm.nih.gov/geo/query/acc.cgi?acc=GSE114412]) and Furuyama (GSE117454 [https://www.ncbi.nlm.nih.gov/geo/query/acc.cgi?acc=GSE117454]). Source data are provided with this paper.

We provide these genesets as a web-based application directly accessible through the website of the human islet research network (https://rapps.hirnetwork.org/scPancMeta).

Field-specific reporting

Please select the one below that is the best fit for your research. If you are not sure, read the appropriate sections before making your selection.

☒ Life sciences ☐ Behavioural & social sciences ☐ Ecological, evolutionary & environmental sciences

For a reference copy of the document with all sections, see nature.com/documents/nr-reporting-summary-flat.pdf

Life sciences study design

All studies must disclose on these points even when the disclosure is negative.

Sample size	For previously published data, full datasets were downloaded without prior filtering whenever possible.
Data exclusions	In single cell RNAseq data, non-islet cells were excluded from differential expression analyses. In GSEA analyses, genesets were excluded if they were smaller than 40 genes, or larger than 500 genes, due to normalization issues.
Replication	After generating identity genes, validation was performed both by literature screening and for selected targets using smFISH or IHC.
Randomization	Data were used as they were available in previously published studies. For our own single cell RNAseq study, 3 donors were used that were selected based on our standard inclusion criteria.
Blinding	Blinding was only applied during quantification of smFISH data, and not required otherwise as all analyses were unsupervised.

Reporting for specific materials, systems and methods

We require information from authors about some types of materials, experimental systems and methods used in many studies. Here, indicate whether each material, system or method listed is relevant to your study. If you are not sure if a list item applies to your research, read the appropriate section before selecting a response.

Materials & experimental systems

n/a	Involved in the study
	☒ Antibodies
☒	Eukaryotic cell lines
☒	Palaeontology and archaeology
☒	Animals and other organisms
	☒ Human research participants
☒	Clinical data
☒	Dual use research of concern

Methods

n/a	Involved in the study
☒	ChIP-seq
	☒ Flow cytometry
☒	MRI-based neuroimaging

Antibodies

Antibodies used

Primary antibodies used were mouse-a-GCG (1:1000, Sigma G2654), goat-a-PPY (1:1000, Novus Biologicals NB100-1793) and rabbita-ARX (1:500, gift from Ken-ichirou Morohashi). The ARX signal was amplified using a donkey-a-rabbit biotin-SP antibody (1:250, Jackson Immunoresearch 711-065-152). Secondary antibodies used were donkey-a-mouse Alexa488 (1:600, Thermo Fisher A21202), donkey-a-goat Alexa568 (1:600, Thermo Fisher A11057) and streptavidin-Alexa647 (1:600, Thermo Fisher S32357).

Validation

The mouse-a-GCG antibody is a regularly used antibody against both murine and human glucagon, for example in pmid 34294685, pmid 30760930, and is considered highly specific for glucagon in pancreatic islets, with limited cross-reactivity in gut glucagon from entero-endocrine cells (not present in the pancreas).

goat-a-PPY: We have publications tested in 2 confirmed species: Human, Mouse. We have publications tested in 5 applications: IB, ICC/IF, IHC, IHC-Fr, IHC-P. Use in Immunocytochemistry/immunofluorescence reported in scientific literature (PMID: 23221614). Use in immunoblotting reported in scientific literature (PMID: 27572106).
rabbit-a-ARX: gifted from Ken-IchirouMorohashi. Published and well-referenced from PMID: 23840809

donkey-a-rabbit biotin SP: Based on immunoelectrophoresis and/or ELISA, the antibody reacts with whole molecule rabbit IgG. It also reacts with the light chains of other rabbit immunoglobulins. No antibody was detected against non-immunoglobulin serum proteins. The antibody has been tested by ELISA and/or solid-phase adsorbed to ensure minimal cross-reaction with bovine, chicken, goat, guinea pig, syrian hamster, horse, human, mouse, rat and sheep serum proteins, but it may cross-react with immunoglobulins from other species.

Thermo Fisher Alexa ABs: To minimize cross-reactivity, these donkey anti-mouse IgG whole antibodies have been affinity-purified and show minimum cross-reactivity to bovine, chicken, goat, guinea pig, hamster, horse, human, mouse, rat, and sheep serum proteins.

Human research participants

Policy information about studies involving human research participants

Population characteristics

SAMN11633049 male age 48 BMI 38.8 non-diabetic, SAMN11963659 male age 52 BMI 32.6 non-diabetic, SAMN12227196 male age 51 BMI 31.8 non-diabetic

Recruitment

Human pancreatic islets from anonymized deceased donors were purchased and obtained through the NIDDK's Integrated Islet Distribution Program (IIDP), which provides islets for fundamental research worldwide (NIH Grant no. DK098085).

Ethics oversight

This type of investigation is outside the scope of the Swiss Human Research Act, and does not require approval by the IRB.

Note that full information on the approval of the study protocol must also be provided in the manuscript.

Flow Cytometry

Plots

Confirm that:

☒ The axis labels state the marker and fluorochrome used (e.g. CD4-FITC).

☒ The axis scales are clearly visible. Include numbers along axes only for bottom left plot of group (a 'group' is an analysis of identical markers).

☒ All plots are contour plots with outliers or pseudocolor plots.

☒ A numerical value for number of cells or percentage (with statistics) is provided.

Methodology

Sample preparation

Human islets from 3 independent donors were dissociated and labeled with cell-surface antibody as previously described in:

Bramswig, N. C. et al. Epigenomic plasticity enables human pancreatic alpha to beta cell reprogramming. J Clin Invest 123, 1275-1284, doi:10.1172/JCI66514 (2013).

Dorrell, C. et al. Human islets contain four distinct subtypes of beta cells. Nat Commun 7, 11756, doi:10.1038/ncomms11756 (2016).

Furuyama, K. et al. Diabetes relief in mice by glucose-sensing insulin-secreting human alpha-cells. Nature 567, 43-48, doi:10.1038/s41586-019-0942-8 (2019).

Instrument	Cells were sorted on a Moflo Astrios (Beckman Coulter) system
Software	Cytomation Summit software
Cell population abundance	For gamma- and epsilon-cell enrichment, all CD9+ cells were complemented to 15’000 cells with CD9- cells. For delta-cell enrichment, 15’000 CD9+ and SSC-HLOW cells were sorted.
Gating strategy	Populations gated in HIC3-2D12 vs. HIC1-2B4 plots were further gated in CD9 vs SSC-H plots.

☒ Tick this box to confirm that a figure exemplifying the gating strategy is provided in the Supplementary Information.
