# Supplementary material for: Generation of human islet cell type-specific identity genesets
Source: Nat Commun. 2022 Apr 19;13:2020. doi: 10.1038/s41467-022-29588-8 (PMC9019032; doi:10.1038/s41467-022-29588-8)
Supplement: Supplementary file 4 — Description of additional supplamental files [file 41467_2022_29588_MOESM4_ESM.docx]

**Supplemental data 1:** Aggregated identity genes for alpha cells. Differential expression analysis for each individual dataset is provided, and a summarized overview is presented to organize the genes.

**Supplemental data 1:** Aggregated identity genes for beta cells. Differential expression analysis for each individual dataset is provided, and a summarized overview is presented to organize the genes.

**Supplemental data 1:** Aggregated identity genes for gamma cells. Differential expression analysis for each individual dataset is provided, and a summarized overview is presented to organize the genes.

**Supplemental data 1:** Aggregated identity genes for delta cells. Differential expression analysis for each individual dataset is provided, and a summarized overview is presented to organize the genes.

**Supplemental data 5:** Genes regulated in the new dataset as presented using pairwise differential expression analysis.

**Supplemental data 6:** Identity genesets with genes annotated for human and mouse gene symbols

**Supplemental data 7:** Details on ortholog conversion going from human to mouse genes for all genes in the identity genesets. Color code provides reference when a human gene is represented by multiple genes in mouse, or when no ortholog is known.

**Supplemental data 8:** Probe sequences used for smFISH
